# The Reservoir Adaptability and Oil Displacement Mechanism of Polymer Microspheres

**DOI:** 10.3390/polym12040885

**Published:** 2020-04-11

**Authors:** Jianbing Li, Liwei Niu, Wenxiang Wu, Meifeng Sun

**Affiliations:** 1 Laboratory of Enhanced Oil Recovery of Education Ministry, Northeast Petroleum University, Daqing 163318, China; lijianbing@vip.sina.com (J.L.); wuwenx@aliyun.com (W.W.); 2 No. 8 Production Plant, Daqing Oilfield Company Limited, Daqing 163514, China; sunmeifeng@petrochina.com.cn

**Keywords:** polymer microspheres, adaptability, matching factor, migration characteristics, in-depth profile control and oil displacement

## Abstract

Polymer microsphere profile control is a promising approach for the profile control of heterogeneous reservoirs. Matching between polymer microspheres and the reservoir pore throat is crucial for profile control. In this study, the range of the optimal matching factor Ra between polymer microspheres and core porosity was divided through core permeability limit experiments, and the dynamic migration laws and shut-off patterns of microspheres were studied using 9-m-long cores and microscopic models. The oil displacement effect and mechanism of microspheres were analyzed using three cores in parallel. The “injectability limit” and “in-depth migration limit” curves were divided by Ra into three zones: blockage (R_a_ < 1.09 ± 0.10), near-well profile control (1.09 ± 0.10 < R_a_ < 5.70 ± 0.64), and in-depth fluid diversion (R_a_ > 5.70 ± 0.64). During migration in porous media, the microspheres gradually enlarged in size and thus successively shut off in four forms: multi-microsphere bridging shut-off, few-microsphere bridging shut-off, single-microsphere shut-off, and elastic shut-off. Microspheres with a rational combination of sizes versus those with a single particle size further enhanced reservoir oil recovery under certain reservoir conditions. Through “temporary shut-off–breakthrough–temporary shut-off,” the polymer microspheres were able to change the fluid flow rate and streamlines, mobilize residual oils, and enhance the oil recovery rates.

## 1. Introduction

The exploitation of nonrenewable petroleum is becoming increasingly difficult as the oilfield development time is prolonged. Thus, petroleum workers commonly aim to maximize the reservoir recovery rate. The majority of oilfields worldwide are developed by water flooding, but long-term flooding and eroding destroy the pore structures of oil reservoirs, and thereby enlarge the radii of reservoir pore throats [1,2]. These factors, together with the heterogeneity of reservoirs and the difference in viscosity between oil and water, result in “preferential channels” between the flooding well and producing well. As a result, the excess flood water circulates less efficiently or inefficiently along the preferential channels, which thereby narrows down the sweep extent of the flood water [3] and largely raises the water content in the produced liquids at a late stage. These consequences directly decrease the oilfield recovery ratio, increase energy consumption in production, and cause environmental pollution [4,5].

Thus, the preferential channels between injection wells and production wells not only reduce the waterflood sweep efficiency but also lead to adverse impacts upon subsequent chemical flooding. Polymer gels and other profile control techniques can prevent, to some extent, the flood water from inefficient circulation along the preferential channels [6,7,8,9,10,11,12], but they are effective only for small radii and cannot solve the deep-water breakthrough of reservoirs. Because of a deeper understanding of reservoirs, the use of chemical agents in deep reservoir processing has attracted wide attention. For this reason, preformed particle gels (PPG), polymer microspheres, inorganic gel coatings, and other deep liquid flow-diverting agents have been developed [13,14,15,16,17,18].

Polymer microspheres are well-known for their heat resistance, salt resistance, shear resistance, and strong deep migration ability [19,20]. The polymer microspheres adopted in profile control are mainly nano-sized and micro-sized. For oilfields with severe waterlogging in the main layers and with highly scattered remaining oil, deep reservoirs can be profile-controlled well by using polymer microspheres to directly regulate the nonuniformity of water flooding and to increase the sweep efficiency. To date, the deep profile control technology of polymer microspheres has been studied in the laboratory and successfully applied to obtain increased oil and decreased water in fields [21,22,23].

The reservoir adaptability and in-depth oil displacement mechanism of polymer microspheres have been studied extensively in recent years. All researchers believe that the matching relationship between the size of polymer microspheres and the pore throat of reservoir rocks considerably influences the profile control and oil displacement effect. Zhao et al. [24] and Dai et al. [25] characterized the matching relationship between polymer microspheres and rock core pores by using the matching factor and found that polymer microspheres achieved the best shut-off and in-depth fluid diversion effects only within a certain range of the matching factor. Micron-sized and nano-sized polymer microspheres are suitable for high and low-permeability cores, respectively [26]. In terms of the in-depth oil displacement mechanism, polymer microspheres are elastic and can reach in-depth strata through elastic deformation and breakthrough, thereby achieving in-depth fluid diversion [27,28,29]. Micron-sized polyacrylamide elastic microspheres can resist the water flow by throat plugging through the mechanisms of trapping plugging, stacking plugging, and bridging plugging [30,31].

During the injection of polymer microspheres, the particle diameter changes with the injection time because of their water absorption and swelling properties. However, researchers have rarely considered the swelling properties of polymer microspheres in porous media when investigating the matching factor of polymer microspheres, and they have always characterized the in-depth migration of polymer microspheres in a short migration distance. In this study, the compatibility between polymer microspheres and reservoir pores was investigated by using the matching factor and permeability limit. The dynamic migration law of polymer microspheres in reservoirs was studied by using a 9-m-long artificial rock core. Micro-displacement experiments were used to observe the shut-off forms of polymer microspheres that had been left to expand for different periods of time in core pores. The microsphere fluid diversion effect and the enhanced oil recovery (EOR) micro-mechanism of polymer microspheres were further clarified by an oil displacement experiment with three cores in parallel. This study provides a scientific basis and technical support for the field application of polymer microspheres in-depth profile control and oil displacement technology.

## 2. Experimental Conditions

### 2.1. Materials

**Figure 1:**
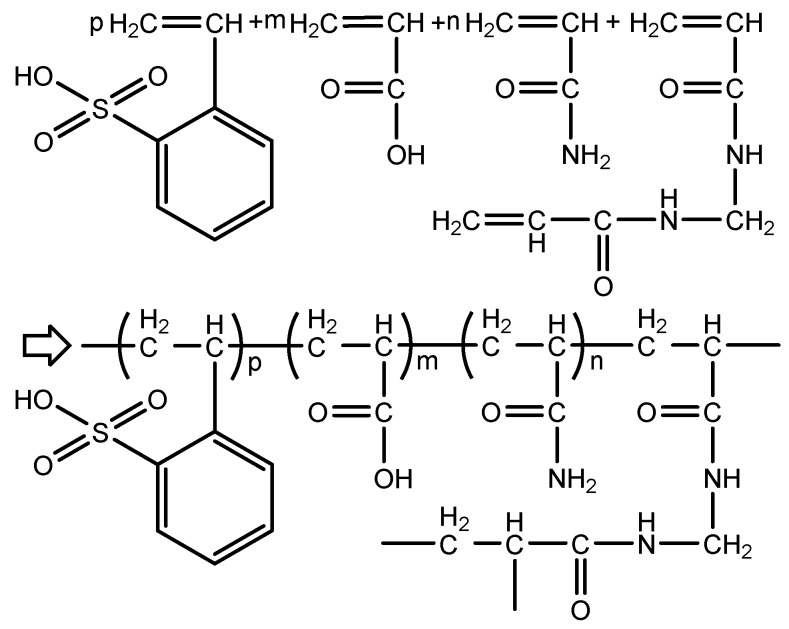
Reaction processes and molecular structures of polymer microspheres.

All 1^#^, 2^#^, and 3^#^ polymer microspheres (PMs) with effective contents of 100% were provided by the Research Institute of Petroleum Exploration Development, Beijing, China. This series of microspheres was prepared using a reversed-phase emulsion method, and the reaction processes and molecular structures are illustrated in Figure 1. Preparation: span 80 (30 portions, g) and tween 80 (3.6 portions, g) were added to 220 portions of white oil and stirred uniformly, forming an oil phase. Then, deionized water (100 portions, g), acrylic acid (AA, Kemiou Chemical Reagent Co., Ltd., Tianjing, China, 10 portions, g), acrylamide (AM, Kemiou Chemical Reagent Co., Ltd., Tianjing, China, 70 portions, g), sodium p-styrenesulfonate (SSS, Bangcheng Chemical Co., Ltd., Tianjing, China, 10 portions, g), and N-N’-methylene bisacrylamide (MBA, Damao Chemical Reagent Factory, Tianjing, China, 0.38 portion, g) were uniformly stirred and adjusted with a NaOH solution to pH 7–8, forming a water phase. After that, the water phase was poured into the oil phase under vigorous stirring. To the resulting homogeneous solution, ammonium persulfate (0.5 portion, g) and sodium bisulfite (0.5 portion, g) were added for the polymerization reaction under nitrogen protection. After stirring at 45 °C for 5 h, the reactants were repeatedly washed with ethanol and then dried for hours at 50 °C, forming dry polymer powders.

Daqing Oilfield formation water and simulated injection water (Table 1) were used to saturate the cores and to prepare polymer microsphere solutions and core displacement, respectively, unless otherwise stated.

**Figure 2:**
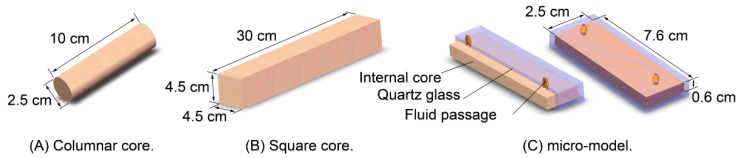
Schematic diagrams of the structures of physical models: (**A**) columnar core; (**B**) square core; (**C**) micro-model.

Artificial homogeneous columnar cores cemented with quartz sand epoxy resin with apparent geometric dimensions of Ø2.5 × 10 cm^2^ (Figure 2A) were used in resistance factor and residual resistance factor experiments [32,33,34]. Artificial homogeneous square cores with apparent geometric dimensions of 4.5 × 4.5 × 30 cm^3^ (H × W × L, the same below) (Figure 2B) were adopted in microsphere fluid diversion effect experiments. A visualized micro-model (0.6 × 2.5 × 7.6 cm^3^, Figure 2C) was used in micro-displacement experiments and consisted of an internal core made of quartz sand and transparent cementitious material and external quartz glass.

**Figure 3:**
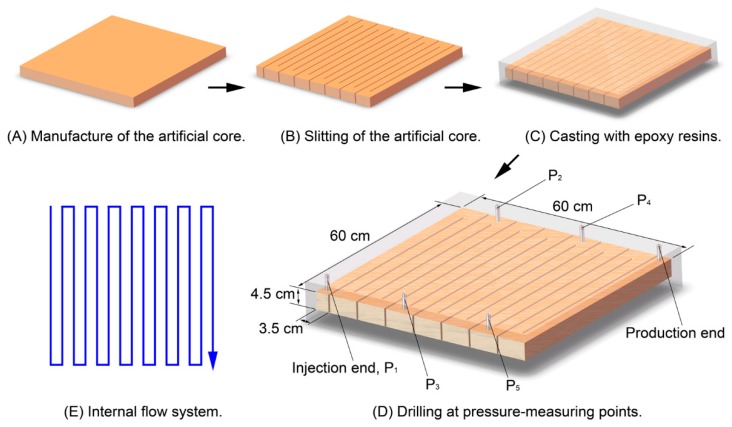
Nine-meter-long core: (**A**) manufacture of the artificial core; (**B**) slitting of the artificial core; (**C**) casting with epoxy resins; (**D**) drilling at pressure-measuring points; (**E**) internal flow system.

The rock core used in the in-depth migration characterization experiments was a 9-m-long artificial core, which was a homogeneous core (length × width × height = 60 × 60 × 4.5 cm^3^) that was processed by slitting and epoxy resin casting [35]. The manufacturing process of the 9-m-long core, illustrated in Figure 3, included the following steps: (A) the artificial homogeneous core (60 × 60 × 4.5 cm^3^) was pressed; (B) the core was slit, as shown in the figure; (C) the slit core was cast with epoxy resin; (D) holes were drilled at the pressure-measuring points. Five pressure-measuring points were distributed evenly in the core along the displacement direction; these points were designated P1 (inlet), P_2_, P_3_, P_4_, and P_5_, and positioned 0, 1.8, 3.6, 5.4, and 7.2 m away from the inlet, respectively. The total length of the fluid flow, starting from the inlet end to the outlet end, was about 9 m (Figure 3E).

### 2.2. Performance Test

Fourier transform infrared (FTIR) spectra of the precursor and polymer microspheres were recorded on a Nicolet Magna-IR 750 FTIR meter (Nicolet, Waltham, MA, USA). Viscosity was measured using an LVDV-Ⅱ+PRO Buchner viscometer (Brookfield Eng. Lab., Stoughton, MA, USA). Particle sizes and distributions were detected on a laser particle analyzer (Zetasizer Nano ZS, Malvern, Worcestershire, UK, Horiba LA-300, Horiba, Kyoto, Japan), and particle sizes are expressed as “x ± s” on the basis of multiple measurements. Micromorphology was observed under an S-3400N scanning electron microscope (Hitachi, Tokyo, Japan) and a SteREO Discovery.V12 microscope (Carl Zeiss, Munich, Germany).

### 2.3. Reservoir Adaptability of Polymer Microspheres

#### 2.3.1. Matching Factor

The matching relationship between polymer microspheres and the core is represented by the matching factor *R_a_*, and the average pore throat diameter can be calculated by the Carman–Kozeny equation. Given that man-made cores are structurally simple, we set $f_{CK}\tau^{2}$ at 4.5 [25] and calculate *R_a_* as follows:

(1) $$R_{a}=\frac{D}{d}=d^{-1}{\left(\frac{72k}{\phi }\right)}^{0.5}$$

where *D* is the average diameter of the core pore throat, μm; *d* is the average particle size of polymer microspheres, μm; *k* is absolute permeability, μm^2^; ϕ is porosity, %; $f_{CK}$ is the Carman–Kozeny form factor; and τ is sinuosity.

#### 2.3.2. Permeability Limit Determination Criterion

The lowest permeability at which the polymer microspheres pass through cores without blockage is called the permeability limit, and it is generally determined by the resistance factor (*F*_R_) and the residual resistance factor (*F*_RR_) [36]. To determine the permeability limit (namely, the fixed particle size) of polymer microspheres, researchers can calculate the *F*_R_ and *F*_RR_ at different matching factors by changing the core permeability. If the core is not blocked, the matching factor (i.e., the core permeability) is continuously decreased by 0.1 until a blockage forms, and the corresponding core permeability is the permeability limit. If the core is blocked, the matching factor is continuously increased by 0.1 until it is unblocked, and the corresponding core permeability is the permeability limit. Usually, the injection pressure changing trend is investigated to determine whether the polymer microspheres block the core. If the polymer microsphere injection pressure constantly rises, it is believed that blockage occurs. In this study, after the limit range of permeability was determined, a rock core with similar permeability was used for experiments. The limit values of permeability are expressed as “x ± s.”

#### 2.3.3. *F*_R_ and *F*_RR_ Test

**Figure 4:**
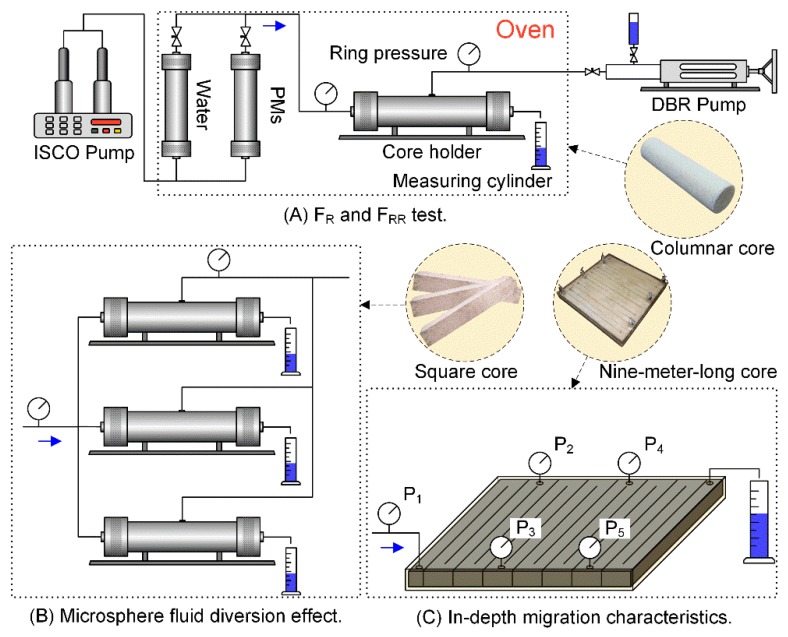
Flow diagram of the core displacement experiment: (**A**) F_R_ and F_RR_ test, (**B**) microsphere fluid division effect, (**C**) in-depth migration characteristics.

The experimental devices mainly included ISCO pumps, pressure sensors, core holders, hand pumps, and vessels. Except for the ISCO pumps, the devices were kept in a constant-temperature chamber at 45 °C. The experimental flow diagram is shown in Figure 4A. The experimental steps were as follows: (1) the core was saturated with water; (2) water flooding was carried out until reaching a stable pressure; (3) 4–6 PV of polymer microspheres was injected; and (4) 4–6 PV of subsequent water was injected.

The experiments were conducted at 45 °C, which was the temperature of Daqing oil reservoirs. A 0.3 wt % polymer microsphere solution was injected at 45 °C, at a rate of 0.3 mL/min.

### 2.4. In-Depth Migration Characteristics

The experimental devices and flow diagram are shown in Figure 4C. The experimental steps were as follows: (1) the model was saturated with water; (2) water flooding was carried out until reaching a stable pressure; (3) 0.2 PV of a 0.3 wt % 2^#^ PM solution was injected into the core at 45 °C at a rate of 0.5 mL/min; and (4) water injection was introduced again until the pressure barely varied. The pressures were recorded, and the pressure gradient between pressure-measuring taps was calculated.

To reduce water swelling before the polymer microsphere solution entered the core, we prepared the solution immediately before injection.

### 2.5. Microscopic Displacement Experiment

**Figure 5:**
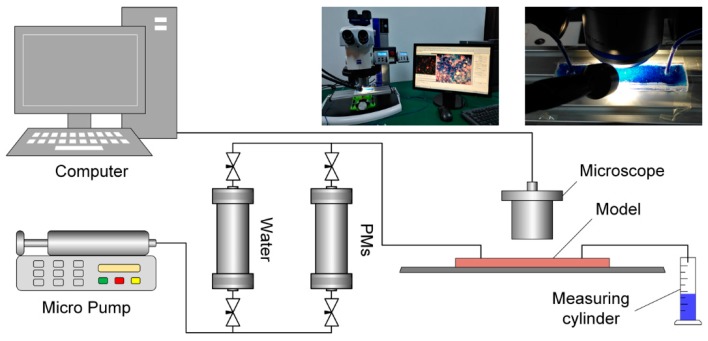
Flow diagram of the microscopic displacement experiment.

The experimental equipment mainly included micro-pumps, intermediate containers, and a SteREO Discovery.V12 stereomicroscope (Carl Zeiss, Germany). The experimental flow diagram is shown in Figure 5. The experimental steps were as follows: (1) the micro-model was saturated with water; (2) a 0.3 wt % solution of 3^#^ polymer microspheres was prepared and dyed with methyl blue; (3) then, the solution was injected into the mold, and the plugging status of the microspheres in the porous medium was observed. The newly prepared polymer microsphere solution was left undisturbed for 1, 3, 5, or 7 days and then used in the experiments.

### 2.6. Microsphere Fluid Diversion Effect

The fluid production and oil production of different permeability layers in different displacement stages were detected by means of injection and separate productions in the model with high, medium, and low-permeability cores in parallel, and thereby used to calculate the fluid production ratio of single layers to whole layers (i.e., diversion percentage). The oil recoveries calculated for the single-layer and whole model aimed to evaluate the fluid diversion capacity of polymer microspheres (core parameters are shown in Table 3). The experimental devices and flow diagram are illustrated in Figure 4B. During the experiments, water flooding was first carried out until reaching a water content of 98%; then, 0.3 PV of a polymer microsphere solution was injected, and water flooding was carried out until reaching a water content of 98%.

Simulated oil with a viscosity of 9.8 mPa·s at 45 °C was mixed using dehydrated oil and kerosene from Daqing Oilfield. After it was left undisturbed for 7 days, the 0.3 wt % polymer microsphere solution was injected into the core at 45 °C at a rate of 0.9 mL/min.

## 3. Results and Discussion

### 3.1. Structures and Performances of Polymer Microspheres

#### 3.1.1. Spectral Analysis

**Figure 6:**
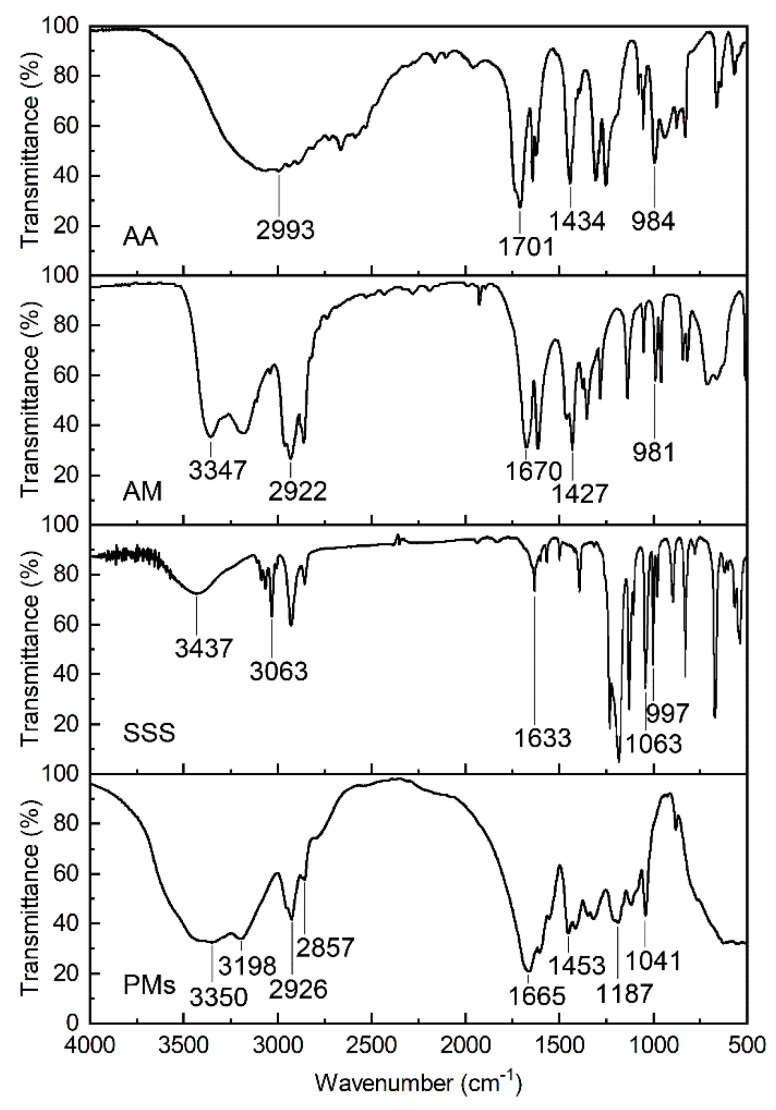
FTIR spectra of the precursor and polymer microspheres.

The FTIR spectra of the precursor and polymer microspheres are illustrated in Figure 6. Clearly, the peaks on curve AA at 2993, 1701, 1434, and 984 cm^-1^ correspond to the stretching vibrations of O–H and C–H in carboxylic acid, the stretching vibration of C=O in the carbonyl group, the vibration of C–H in olefin, and the vibration of –CH=CH_2_ in olefin, respectively. The peaks on curve AM at 3347, 2922, 1670, 1427, and 981 cm^−1^ are ascribed to the stretching vibration of N–H, the vibration of C–H in the alkyl group, the stretching vibration of C=O in the amide group, the vibrations of C–N and N–H in the amide, and the stretching vibration of C=C, respectively. The peaks on curve SSS at 3437, 3063, 1633, 1063, and 997 cm^−1^ are ascribed to the stretching vibrations of O–H and C–H in carboxylic acid, the vibration of C–H in olefin, the vibration of C–H in olefin, the stretching vibration of S=O in sulfonic acid, and the stretching vibration of C=C, respectively. The peaks on curve PMS at 3350, 2926 and 2857, 1665 and 1606, 1453, and 1187 and 1041 cm^−1^ are attributed to the stretching vibrations of O–H and C–H in carboxylic acid and the vibration of N–H in the amino group, the vibration of C–H in the alkyl group, the stretching vibration of C=O, the vibration of C–N and N–H in the amide, and the asymmetric stretching vibration and symmetric stretching of S=O in sulfonic acid, respectively; however, the stretching vibration peaks of C=C disappeared, indicating that the three monomers had polymerized.

#### 3.1.2. Particle Size

**Figure 7:**
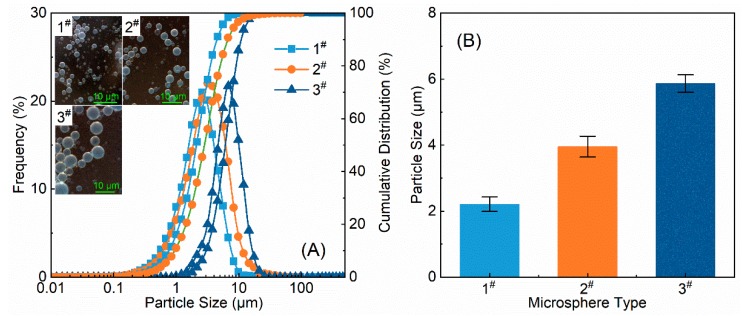
(**A**) Particle size distribution and (**B)** initial particle sizes of polymer microspheres in aqueous solutions.

The measured initial particle size and particle size distribution of polymer microspheres in an aqueous solution are displayed in Figure 7. Clearly, the polymer microspheres dispersed very well in the aqueous solution and were sized between 0.5 and 20 μm (Figure 7A). The particle sizes of 1^#^, 2^#^, and 3^#^ polymer microspheres were 2.208 ± 0.22, 3.952 ± 0.31, and 5.865 ± 0.27 μm, respectively (Figure 7B).

**Figure 8:**
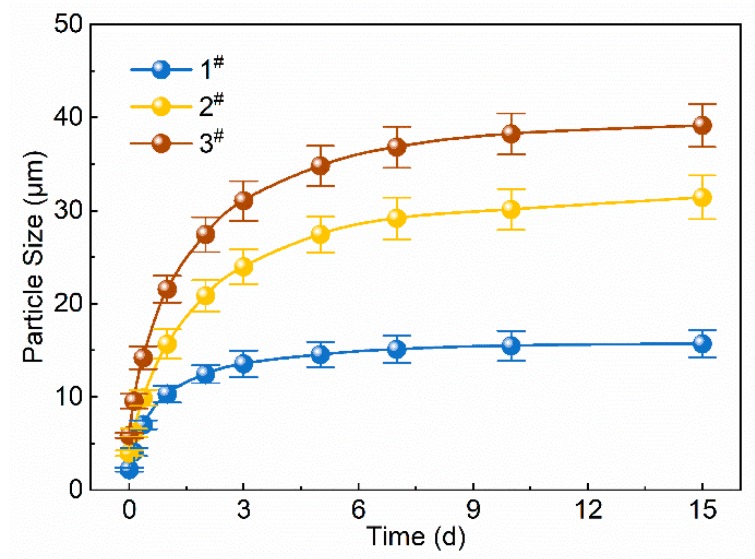
Average particle size vs. expansion time of polymer microspheres in an aqueous solution at 45 °C.

The relationship between average particle size and the expansion time of polymer microspheres in an aqueous solution at 45 °C is shown in Figure 8. Clearly, the particle sizes enlarged rapidly at the initial stage of preparation and then grew slowly after 7 days (Figure 8). On the 7th day, 1^#^, 2^#^, and 3^#^ PMs had average particle sizes of 15.110 ± 1.49, 29.156 ± 2.35, and 36.813 ± 2.33 μm, respectively, which were 6.8 ± 0.36, 7.4 ± 0.32, and 6.3 ± 0.31 times the initial sizes (2.208 ± 0.22, 3.952 ± 0.31, and 5.865 ± 0.27 μm), respectively. This was because the polymer microspheres were cross-linked polyacrylamide gel particles in a three-dimensional network structure, and their enriched free hydrophilic groups (–CONH_2_) on the surface readily combined with polar water molecules to form hydration layers (diffused electric double layers), which comprised the bound water. The combination was fast and short. After the formation of the hydration layers, the polymer network of the microspheres expanded accordingly, and the hydrophilic groups hydrolyzed into free ions, leading to an ion concentration difference between the inside and outside of the polymer network, which is called the osmotic pressure difference. Thus, water molecules permeated into the polymer network as free water. In addition, the formation of hydrogen bonds between free water and hydrophilic groups in the polymer network constantly promoted the hydrolysis of hydrophilic groups and the appearance of an osmotic pressure difference. Consequently, the water continuously entered the polymer network, and the microspheres absorbed a large amount of water and gradually expanded. At the initial stage, the larger osmotic pressure difference led to a faster expansion of elastic microspheres. When the water absorption reached a certain level and the expansion slowed down, the osmotic pressure difference decreased and stabilized. The above data imply that the polymer microspheres can expand to a certain size after enough hydration and effectively block the pore throats of high-permeability zones in the formation.

#### 3.1.3. Micromorphology

**Figure 9:**
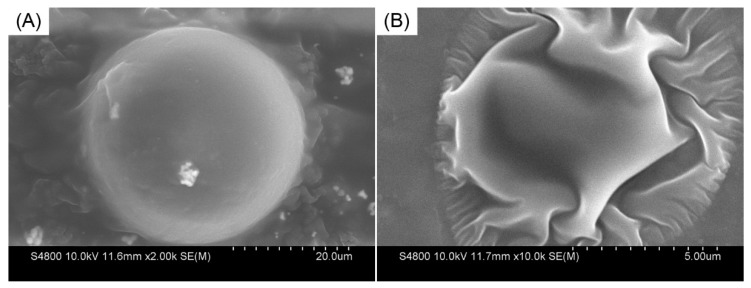
SEM photos of 3^#^ PMs (**A**) expanded for 7 days in an aqueous solution and (**B**) precipitated in alcohol after expansion for 7 days in an aqueous solution.

SEM showed that the 3^#^ PMs that swelled for 7 days at 45 °C were uniform with a clear outline (Figure 9A). After the 3^#^ PMs that swelled for 7 days were filtered and added to ethanol for a period of time, the expanded polymer microspheres dehydrated rapidly and were folded (Figure 9B). The 3^#^ PMs expanded and shrank uniformly, suggesting that the cross-linking points in the 3^#^ PMs were uniformly distributed and isotropic.

### 3.2. In-Depth Migration Characteristics

**Figure 10:**
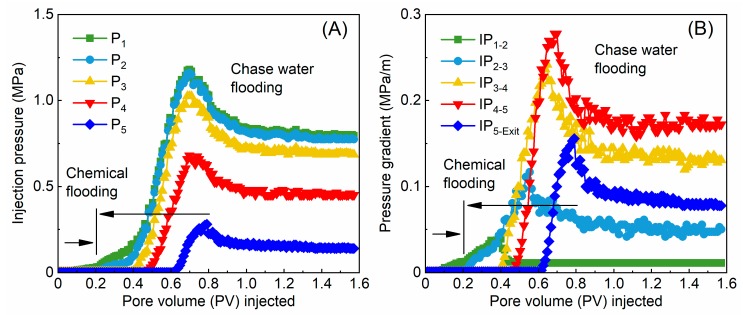
Curves of (**A**) injection pressure vs. PV and (**B**) pressure gradient vs. PV (IP_(m–n)_ represents the pressure gradient between points m and n).

The relationships between the injection pressure *P_x_* (*x* is the number of pressure-measuring taps) and pore volume (PV) and between the pressure gradient and PV are shown in Figure 10. P_1_ and P_2_ both increased in turn at the stage of microsphere injection (Figure 10A). The polymer microspheres had not yet reached P_3_, so P_3_, P_4_, and P_5_ did not change significantly. In the subsequent stage of water flooding, P_1_ and P_2_ further increased. The flood water extracted some polymer microspheres from the cores, so the injection pressure decreased and stabilized. Because of the in-depth migration of polymer microspheres in porous media, P_3_, P_4_, and P_5_ were elevated in turn and then decreased and stabilized. The subsequent water flooding stage showed a higher pressure rise than the chemical flooding stage, indicating that the polymer microspheres expanded while migrating in the core and were highly capable of in-depth migration and shut-off.

The pressure gradients between the pressure-measuring taps from the injection end (P_1_) to the production end increased first and then decreased; the pressure gradient between P_4_ and P_5_ was the largest, i.e., IP_(1–2)_ < IP_(2–3)_ < IP_(3–4)_ < IP_(4–5)_ > IP_(5–Exit)_ (Figure 10B). There are mainly four reasons for this observation. (1) The polymer microspheres migrated and expanded in porous media, and their particle sizes in core pores and the seepage resistance were enlarged in the flow direction, which increased the pressure gradient. (2) Continuous retention in the flow direction led to a decrease in polymer microsphere concentration and a decrease in the pressure gradient in the flow direction. (3) The polymer microspheres expanded at a decreasing rate over time and were damaged by shear more readily, thereby slowing the pressure gradient increase caused by the expansion of polymer microspheres. (4) The extraction of polymer microspheres also led to a significant decrease in the pressure gradient. The pressure gradients between the pressure-measuring taps in the cores resulted from the joint interaction of the above aspects.

### 3.3. Shut-Off Forms of Polymer Microspheres in the Core Pore

**Figure 11:**
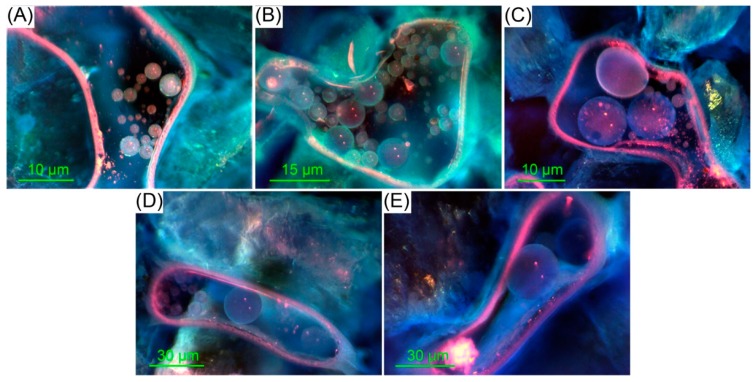
Shut-off forms of polymer microspheres in the core pore after swelling times of (**A**) 0, (**B**) 1, (**C**) 3, (**D**) 5, and (**E**) 7 days.

The 3^#^ PM solution at varying swelling times was used in the micro-displacement experiments in order to study the micro-shut-off form of polymer microspheres that swelled to different particle sizes in porous media (Figure 11). The polymer microspheres had a small particle size in the initial stage of preparation, and they could mostly pass through the core pore smoothly, which complicated the effective shut-off (Figure 11A). After the polymer microspheres swelled in an aqueous solution for 1 day, their particle size increased, leading to multi-particle bridging plugging in the core (Figure 11B). The probability of multi-particle bridging was low unless a large number of microspheres worked together. With the prolonging of the swelling time, their particle size was further enlarged, and a decreasing number of microspheres could block the core pore throat by bridging (Figure 11C), leading to a gradual increase in shut-off probability. When their diameter rose to the size of the core pore, a single microsphere could block the core pore throat and reduce the flow resistance (Figure 11D). When the diameter of the polymer microspheres surpassed that of the core pore, they elastically deformed and thereby blocked the core pore throat (Figure 11E). Then, after further elastic deformation, the polymer microspheres broke through the pore throat at a certain displacing force, quickly restored their original shape and size, and further migrated under the action of fluid flow because of their physicochemical properties. The polymer microspheres achieved in-depth profile control and oil displacement through elastic shut-off → elastic deformation → stable migration → elastic recovery → shut-off when their particle size matched the core permeability.

### 3.4. The Reservoir Adaptability of Polymer Microspheres

#### 3.4.1. Permeability Limit

The measured *F*_R_ and *F*_RR_ of the polymer microspheres that were newly prepared or swelled for 7 days are listed in Table 2. For polymer microspheres of the same type and with the same swelling time, the *F*_R_ and *F*_RR_ increased with decreasing core permeability (Table 2). When the core permeability declined to a certain level, the core pore throat was blocked. The 1^#^, 2^#^, and 3^#^ PMs that were newly prepared and swelled for 7 days had permeability limits of 15.4 ± 2.6 × 10^−3^ and 708.4 ± 18.7 × 10^−3^, 63.8 ± 9.3 × 10^−3^ and 2755.5 ± 41.9 × 10^−3^, and 134.3 ± 17.5 × 10^−3^ and 4285.7 ± 154.8 × 10^−3^ μm^2^, respectively.

**Figure 12:**
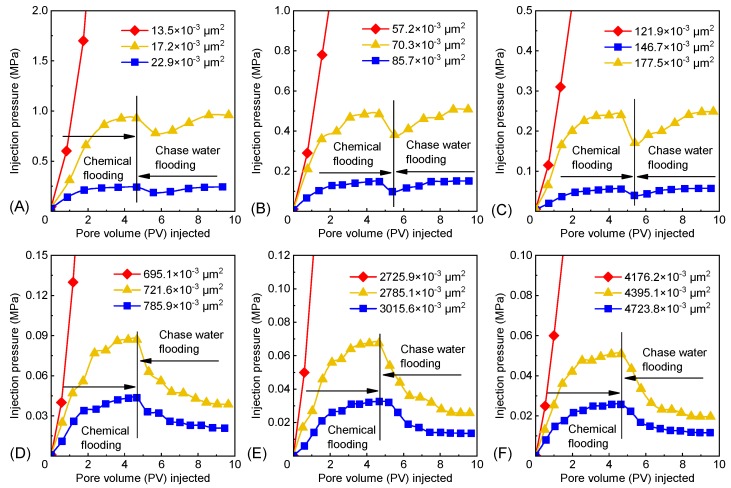
Injection pressure vs. pore volume (PV) injected: (**A**,**B**,**C**) 1^#^, 2^#^, and 3^#^ PMs that were newly prepared; (**D**,**E**,**F**) 1^#^, 2^#^, and 3^#^ PMs that swelled for 7 days.

The relationship between the injection pressure and pore volume (PV) injected is illustrated in Figure 12. With an increasing PV at a high core permeability during chemical flooding, the injection pressure of polymer microspheres rose and plateaued (Figure 12). For a low core permeability, as PV increased, the injection pressure was elevated, indicating that the injection of the polymer microspheres was very difficult; i.e., blockage occurred.

#### 3.4.2. Rational Matching Factor

**Figure 13:**
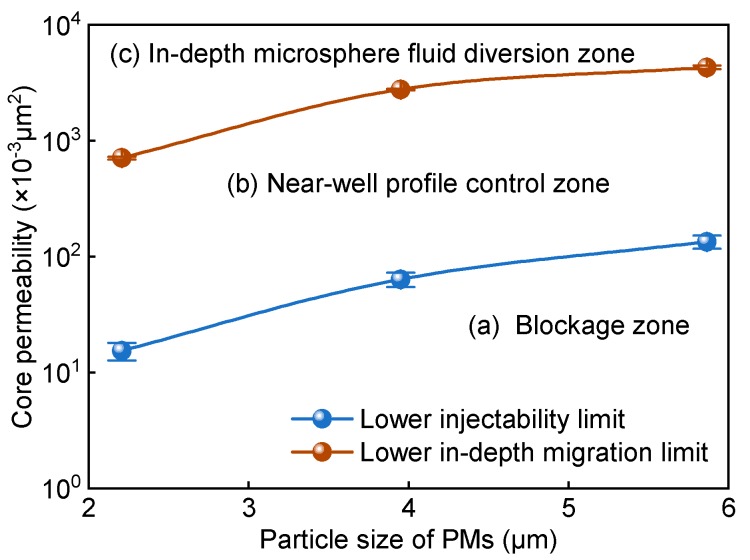
Core permeability vs. particle size of polymer microspheres.

According to the experimental results in Table 2, the relationship between the core permeability and the particle size of polymer microspheres can be determined (Figure 13). The y-axes (permeability) of the “lower injectability limit” curve and “lower in-depth migration limit” curve are the permeability limits of polymer microspheres that were newly prepared and swelled for 7 days, respectively (Figure 13). The two curves divide the coordinates into three zones: a blockage zone (a), a near-well profile control zone (b), and an in-depth microsphere fluid diversion zone (c). In zone a, the polymer microspheres, which could not be injected into the core because their size exceeded that of the core pore, accumulated and blocked the core. In zone b, the fresh polymer microspheres could be smoothly injected into the core, but the polymer microspheres that swelled to a certain level blocked the pore and were unable to migrate to deep strata. Thus, the polymer microspheres in zone b were used only for profile control in the near-well-bore area. In zone c, both the fresh and the swelled polymer microspheres could migrate in porous media into deep strata for in-depth microsphere fluid diversion. With further increases in the core permeability, the in-depth fluid diversion capacity of the polymer microspheres was further enhanced. When the core permeability rose to a certain level, however, both the shut-off capacity and the microsphere fluid diversion of the polymer microspheres were weakened. Thus, polymer microspheres provide the optimal in-depth fluid diversion effect only within a certain range of reservoir permeability.

**Figure 14:**
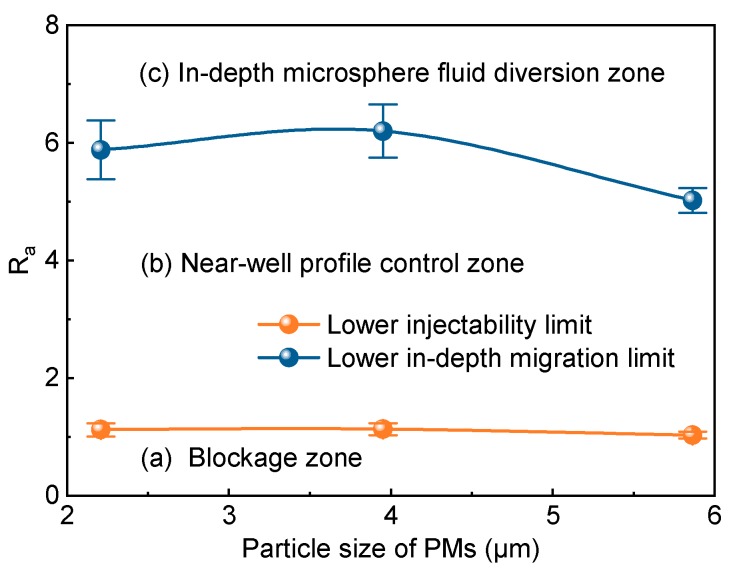
Matching factor Ra vs. particle size of polymer microspheres.

According to the definition of the matching factor R_a_, Figure 13 is converted to the relationship between R_a_ and the particle size of the polymer microspheres (Figure 14, in which R_a_ is the diameter ratio of the rock pore throat to fresh polymer microspheres). The polymer microspheres with different particle sizes had similar optimal matching factors (Figure 12). The lower injectability limits of 1^#^, 2^#^, and 3^#^ PMs correspond to matching factors of 1.12 ± 0.11, 1.13 ± 0.10, and 1.03 ± 0.06, respectively, with an average of 1.09 ± 0.10. The lower in-depth migration limits of 1^#^, 2^#^, and 3^#^ PMs correspond to matching factors of 5.88 ± 0.50, 6.20 ± 0.45, and 5.02 ± 0.21, respectively, with an average of 5.70 ± 0.64. Therefore, polymer microspheres are difficult to inject into strata at *R*_a_ < 1.09 ± 0.10 (zone a), suitable for profile control in the near-well-bore area at 1.09 ± 0.10 < *R*_a_ < 5.70 ± 0.64 (zone b), and capable of in-depth fluid diversion at *R*_a_ > 5.70 ± 0.64 (zone c).

### 3.5. The Microsphere Fluid Diversion Effect and Oil Displacement Mechanism of Polymer Microspheres

The oil recovery and enhanced oil recovery (EOR) after the profile control and flooding of polymer microspheres are summarized in Table 3. Scheme 2 (18.2%) has a 7.8% higher EOR than Scheme 1 (10.4%) at the same polymer microsphere consumption (Table 3). Schemes 1 and 2 are only slightly different in terms of EOR in medium-/high-permeability layers. Scheme 2 (36.8%) has a significantly higher EOR (an increase of 32.1%) in low-permeability layers than Scheme 1 (only 4.7%). Thus, the difference in EOR mainly depends on the utilization degree of the remaining oil in the low-permeability layer. The above data suggest that microspheres with a rational combination of particle sizes, compared with those with a single particle size, are more capable of greatly enhancing the oil recovery from reservoirs under certain reservoir conditions.

**Figure 15:**
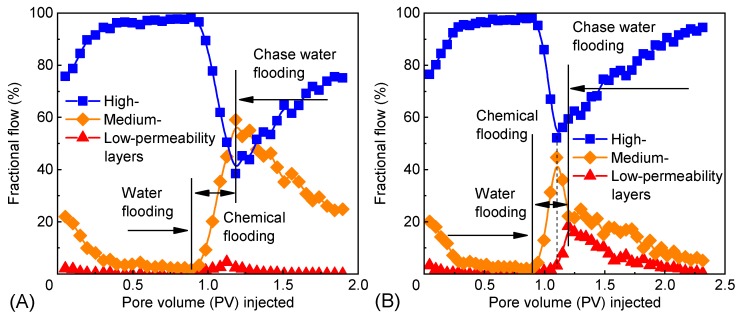
Fractional flow and pore volume (PV) injected in each layer: (**A**) scheme 1; (**B**) scheme 2.

The relationship between the diversion rate and the pore volume (PV) injected in the experiment is illustrated in Figure 15. In the stage of water flooding, the high-permeability layer had the lowest suction threshold pressure, the highest suction pressure differential (the gap between the injection pressure and suction threshold pressure), and the largest fluid absorption capacity and diversion rate. Similarly, the low-permeability layer had the highest suction threshold pressure and the lowest diversion rate. In the stage of chemical flooding, the polymer microspheres first entered, resided in, and shut off the high-permeability layer; reduced the flow cross-section of pores; and increased the flow resistance, forcing the medium and low-permeability layers to absorb the fluid. In the subsequent water flooding step, with the extraction of polymer microspheres, the diversion rate of the high-permeability layer rose again, while the diversion of medium and low-permeability layers decelerated.

The polymer microspheres that swelled for 7 days could be smoothly injected into the core only when the matching factor was in zone c (Figure 14). In Scheme 1 (Table 3), the matching factor between 3^#^ PMs and the high-permeability layer is 5.29 ± 0.23, which is in zone c (Figure 14), so 3^#^ PMs can be smoothly injected into the high-permeability layer. The matching factor between 3^#^ PMs and the medium-permeability layer is 2.39 ± 0.11, which is in zone b (Figure 14). This indicates that 3^#^ PMs are difficult to inject into the medium-permeability layer. Therefore, 3^#^ PMs mainly entered the high-permeability layer of the core and adjusted the fluid entry profile between medium and high-permeability layers. However, the fluid entry profile between low and medium-permeability layers was not effectively adjusted, so the diversion of the low-permeability layer accelerated slightly (Figure 15A). In addition, the difference in permeability between medium and low-permeability layers was the main barrier to further enhancing the oil recovery.

On the basis of injecting 0.2 PV of 3^#^ PMs, another 0.1 PV of 1^#^ PMs was injected in Scheme 2 (Table 3). The matching factors between 1^#^ PMs and the high-permeability layer and between 1^#^ PMs and the medium-permeability layer are 14.24 ± 1.36 and 6.34 ± 0.60, respectively, which are both in zone c (Figure 14), so 1^#^ PMs can be smoothly injected into both the medium and high-permeability layers of the core. However, the matching factor between 1^#^ PMs and the low-permeability layer is 2.44 ± 0.23, which is in zone b (Figure 14), so it is difficult to inject 1^#^ PMs into the low-permeability layer. On the basis of the 3^#^ PM-adjusted fluid entry profile of medium and high-permeability layers, 1^#^ PMs can enter the medium-permeability layer in addition to the high-permeability layer to adjust the fluid entry profile between low and medium-permeability layers, and thus greatly accelerate the diversion of the low-permeability layer (Figure 15B).

**Figure 16:**
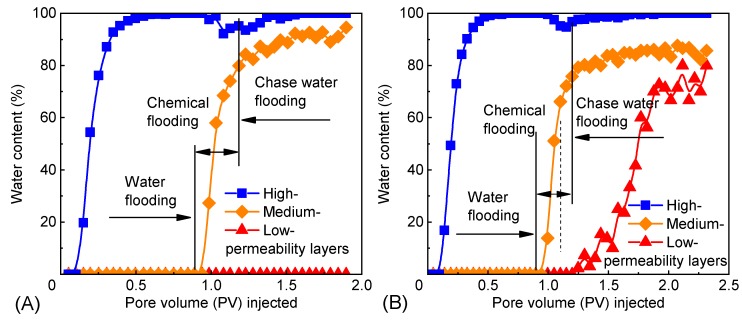
Water content and pore volume (PV) injected in each layer: (**A**) scheme 1; (**B**) scheme 2.

The relationship between the water cut of each layer and the pore volume (PV) injected in the experiment is displayed in Figure 16. The water cut of the high-permeability layer was close to 100% in the late stage of water flooding, but after the injection of microspheres, the water cut decreased to a certain extent, and the oil recovery rose to some degree (Table 3). Scheme 1 (Table 3) had more 3^#^ PMs injected into the high-permeability layer and a higher EOR from the high-permeability layer than Scheme 2 (Table 3). This indicates that in addition to profile control, the polymer microspheres can also, to some extent, mobilize the residual oil and decrease the oil saturation.

**Figure 17:**
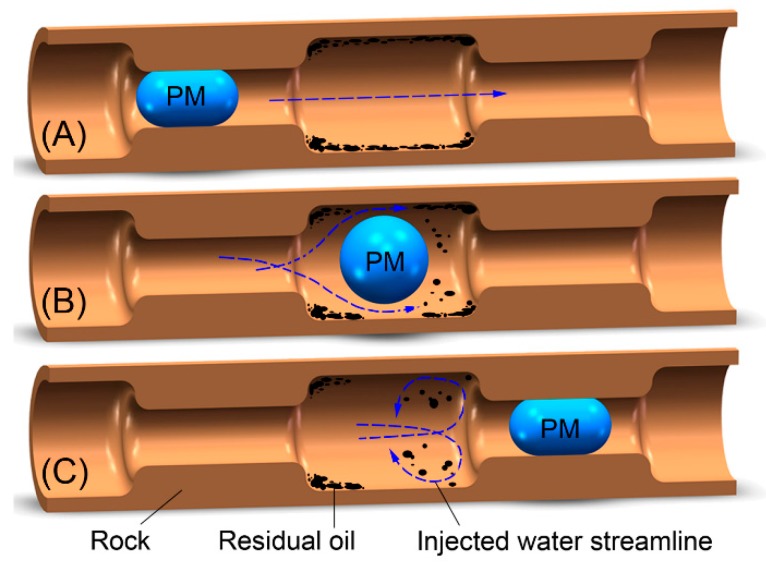
Migration of polymer microspheres in the core pore: polymer microspheres (**A**) temporarily shut off a narrow and small pore; (**B**) break through the small pore and enter a big pore; (**C**) temporarily shut-off a small pore.

Figure 17 shows the migration of polymer microspheres in core pores. In the larger pore spaces, the fluid in the center flowed faster, and the fluid closer to the edge flowed more slowly, so the fluid imposed a small force on the residual oil on the surface of the rock pore. The polymer microspheres in a narrow pore throat elastically deformed, and the injected water moved forward slowly (Figure 17A). The polymer microspheres broke through the narrow pore throat and instantaneously entered the large pore throat at a certain displacing force, so the microspheres and fluids flowed at an instantaneously increasing rate in the large pore spaces. In addition, the fluid streamline in the large pore throats shifted from the center to the edge of the pore throats. The faster-flowing fluid struck the residual oil on the surface of the rock pore, so the residual oil was removed from the surface of the rock pore and recovered along with the fluid (Figure 17B). When the polymer microspheres entered narrow and small pore throats again, the fluid flowed at an instantaneously decreasing rate, the fluid in the original large pore spaces flowed out of disorder, and some of the fluid acted on the residual oil at the junction of the large and small core pores, so some residual oil was removed and recovered along with subsequent fluid (Figure 17C). The polymer microspheres mainly utilized the residual oil through the above two actions to enhance the oil recovery from reservoirs, but the EOR was very limited.

In summary, the EOR mechanism is that the polymer microspheres control the fluid entry profiles of heterogeneous reservoirs and expand the injected water-swept volume. The polymer microspheres also play a positive role in the utilization of residual oil through temporary shut-off → breakthrough → temporary shut-off.

## 4. Conclusions

The particle size and distribution of polymer microspheres in water were characterized, and the micro-morphology was observed. The dynamic migration law of polymer microspheres was studied through 9-m-long core displacement experiments. The shut-off forms of polymer microspheres with different swelling times in porous media were observed through micro-displacement experiments. The optimal matching factors between polymer microspheres and the core pore were classified through fluidity experiments. The profile control and flooding effect and oil displacement mechanism were analyzed through an oil displacement experiment of three cores in parallel.

1. The three kinds of micrometer-sized polymer microspheres had water absorption and swelling properties, swelled at a decreasing rate after 7 days, and expanded to 6–8 times their initial particle sizes.
2. When the polymer microspheres migrated in porous media, their particle sizes gradually enlarged and thus successively shut off in four forms: multi-microsphere bridging shut-off, few-microsphere bridging shut-off, single-microsphere shut-off, and elastic shut-off. The pressure gradient between pressure-measuring taps along the flow direction increased first and then decreased.
3. The injectability limit and in-depth migration limit of polymer microspheres were decreased through core permeability limit tests. These two curves divided core permeability into a blockage zone (R_a_ < 1.09 ± 0.10), a near-well profile control zone (1.09 ± 0.10 < R_a_ < 5.70 ± 0.64), and an in-depth microsphere fluid diversion zone (R_a_ > 5.70 ± 0.64).
4. Microspheres with a rational combination of particle sizes were more capable of enhancing the oil recovery from reservoirs than those with a single particle size under certain reservoir conditions.
5. From small pore throats to large pore throats, the polymer microspheres, through “temporary blocking-breaking through,” changed the flow lines of fluids in large pores and enlarged the flow velocity, thereby mobilizing the residual oils. From large pore throats to small pore throats, the polymer microspheres, through “breaking through-temporary blocking,” subjected the fluid flows in large pore throats to chaos and peeled off some of the residual oils, thereby improving the oil recovery rate.

## Figures and Tables

**Table 1:** Water property analysis.

Parameter	Positive Ions (mg/L)			Negative Ions (mg/L)				Total Mineral Content (mg/L)
	Na^+^	Ca^2+^	Mg^2+^	HCO_3_^−^	CL^–^	SO_4_^2–^	CO_3_^2–^	
Formation water	2428.00	14.90	7.48	2160.08	2266.88	54.10	197.66	7156.5
Injection water	1265.00	32.10	7.30	1708.56	780.12	9.61	210.07	4012.7

**Table 2:** *F*_R_ and *F*_RR_ of polymer microspheres that were newly prepared and swelled for 7 days.

Microsphere Type	Swelling Time (days)	Work Viscosity (mPa·s)	Test Type	Core Parameter			Matching Factor R_a_ = D/d	F_R_	F_RR_
				Effective Permeability *K*_W_ (×10^−3^ μm^2^)	Porosity *ϕ* (%)	Average Throat Diameter of Cote, *D* (μm)			
1^#^	0	1.4	Screening	13.5	17.72	2.34	1.07 ± 0.10	Blockage	-
			Repetition	13.2	17.70	2.32	1.06 ± 0.10	Blockage	-
			Screening	17.2	18.73	2.57	1.17 ± 0.11	25.5	26.4
			Repetition	16.8	18.75	2.54	1.16 ± 0.11	25.6	26.4
			Screening	22.9	19.31	2.92	1.33 ± 0.13	8.8	8.9
	7	2.7	Screening	695.1	30.59	12.79	5.83 ± 0.56	Blockage	-
			Repetition	697.6	30.57	12.82	5.84 ± 0.56	Blockage	-
			Screening	721.6	30.69	13.01	5.93 ± 0.56	100.6	44.6
			Repetition	712.3	30.64	12.94	5.90 ± 0.56	103.7	45.1
			Screening	785.9	31.21	13.46	6.14 ± 0.58	54.8	26.2
2^#^	0	1.6	Screening	57.2	22.81	4.25	1.08 ± 0.09	Blockage	-
			Repetition	58.9	22.81	4.31	1.10 ± 0.09	Blockage	-
			Screening	70.3	23.81	4.61	1.17 ± 0.09	54.5	57.2
			Repetition	70.5	23.79	4.62	1.17 ± 0.09	54.8	57.4
			Screening	85.7	24.89	4.98	1.27 ± 0.10	20.2	20.7
	7	3.1	Screening	2725.9	33.57	24.18	6.14 ± 0.49	Blockage	-
			Repetition	2729.3	33.51	24.22	6.15 ± 0.50	Blockage	-
			Screening	2785.1	33.16	24.59	6.25 ± 0.50	300.8	114.8
			Repetition	2768.1	33.15	24.52	6.23 ± 0.50	289.1	107.5
			Screening	3015.6	33.28	25.54	6.49 ± 0.52	156.9	65.1
3^#^	0	1.7	Screening	121.9	25.97	5.81	0.99 ± 0.04	Blockage	-
			Repetition	117.3	25.89	5.71	0.97 ± 0.04	Blockage	-
			Screening	146.7	26.52	6.31	1.08 ± 0.05	56.2	58.3
			Repetition	151.6	26.53	6.41	1.09 ± 0.05	54.1	57.9
			Screening	177.5	26.61	6.93	1.18 ± 0.84	15.7	16.2
	7	3.8	Screening	4176.2	35.42	29.14	4.98 ± 0.22	Blockage	-
			Repetition	4187.8	35.41	29.18	4.98 ± 0.22	Blockage	-
			Screening	4395.1	35.82	29.72	5.07 ± 0.22	358.1	138.1
			Repetition	4378.2	35.80	29.67	5.07 ± 0.22	350.3	127.9
			Screening	4723.8	35.93	30.77	5.25 ± 0.23	194.8	88.1

**Table 3:** Oil recovery and enhanced oil recovery after polymer microsphere profile control and flooding.

Scheme No.	PM Type and Injection	Layer	Core Parameter			R_a_ = D/d		Oil Saturation(%)	Oil Recovery (%)		
			*K*_W_ (×10^−3^μm^2^)	Porosity (%)	D (μm)	3^#^ PMs	1^#^ PMs		Water Flooding	Chemical Flooding	Added Value
1	3^#^ PMs (0.3PV)	K_3_	107.2	25.32	5.52	0.94 ± 0.04	-	64.7	1.7	6.4	4.7
		K_2_	817.9	30.13	13.98	2.39 ± 0.11	-	72.6	23.9	47.0	23.1
		K_1_	4482.6	33.61	30.99	5.29 ± 0.23	-	81.2	51.4	55.0	3.6
		All the layers	1802.6	-	-	-	-	73.6	29.8	40.2	10.4
2	3^#^ PMs (0.2PV) + 1^#^ PMs, (0.1PV)	K_3_	101.7	25.46	5.36	0.92 ± 0.04	2.44 ± 0.23	63.4	2.0	38.8	36.8
		K_2_	825.4	30.67	13.92	2.38 ± 0.11	6.34 ± 0.60	72.8	21.8	44.5	22.7
		K_1_	4496.1	33.14	31.25	5.34 ± 0.24	14.24 ± 1.36	81.6	51.7	55.0	3.3
		All the layers	1807.7	-	-	-	-	73.4	29.2	47.4	18.2

^3^ K_1_, K_2_, and K_3_ stand for high, medium, and low-permeability layers, respectively.
